# Safety of Supraclavicular Approach for Dual-Lumen Catheter Placement in Veno-Venous Extracorporeal Membrane Oxygenation: A Case Report

**DOI:** 10.7759/cureus.59033

**Published:** 2024-04-25

**Authors:** Yuichi Horikoshi, Jun Hamaguchi, Kengo Asano, Takeo Matsuyoshi, Keiki Shimizu

**Affiliations:** 1 Department of Emergency and Critical Care Medicine, ECMO (Extracorporeal Membrane Oxygenation) Center, Tokyo Metropolitan Tama Medical Center, Tokyo, JPN

**Keywords:** configuration change, supraclavicular approach, avalon, dual-lumen catheter, v-v ecmo

## Abstract

In veno-venous extracorporeal membrane oxygenation (V-V ECMO), the dual-lumen catheter (DLC) facilitates mobility, reduces recirculation, and mitigates the risk of infection. The right internal jugular vein (IJV) is the most common site for DLC insertion. Still, it is often unavailable for various reasons, including local infection, hematoma, or thrombus. A 64-year-old male patient with mantle lymphoma, which was in remission after autogenous blood transplantation, suffered lung damage and refractory pneumothorax from coronavirus disease 2019 (COVID-19) and required V-V ECMO treatment initiated on day 39. The patient was unable to be weaned off V-V ECMO due to uncontrolled high serum carbon dioxide (CO_2_) concentration and required long-term V-V ECMO treatment for more than 80 days. DLC placement was necessary to implement aggressive rehabilitation, reduce puncture site-induced infections, and reduce recirculation. On day 119, a supraclavicular approach was used for DLC placement under fluoroscopic guidance using ultrasound guidance because a thrombus in the right IJV prevented the DLC insertion at a usual puncture site. Rehabilitation was safely performed at a higher intensity than preoperatively of DLC insertion. Overall, the DLC catheter was maintained for more than 30 days until the patient died due to septic shock by an unknown focus on day 150, with no complications such as bleeding or infection. This case report highlights the significance of using the supraclavicular approach for DLC placement in V-V ECMO in cases where IJV is not possible due to thrombus presence. In conclusion, the supraclavicular approach is safe and feasible for V-V ECMO insertion as an alternative to the IJV.

## Introduction

Veno-venous extracorporeal membrane oxygenation (V-V ECMO) is an invaluable method of respiratory therapy in adult cases of respiratory failure that are refractory to mechanical ventilation. The use of V-V ECMO as a supportive therapy in severe respiratory failure has increased increasingly over the past decade. V-V ECMO can temporarily provide adequate oxygen to systemic organs as a bridge to respiratory support until autologous lung function is restored. Conventional V-V ECMO uses two cannulas, which leaves the patient immobile and requires sedation and mechanical ventilation in most cases. Recently, the dual-lumen catheter (DLC) has come to be used in V-V ECMO to facilitate mobilization (avoid femoral cannulation) by freeing the femoral vein and the risk of infection from additional insertions. In addition, the DLC catheter allows simultaneous removal of deoxygenated blood from both the superior vena cava (SVC) and inferior vena cava (IVC) and return of oxygenated blood to the right atrium (RA) to minimize recirculation. Its disadvantage, however, is that the insertion sites are limited, especially when the internal jugular vein (IJV) is unavailable due to a vascular malformation, stenosis, or thrombosis, for instance. The inability to insert the DLC in these situations is detrimental to the patient, and a site where the DLC can be safely inserted instead of the right IJV should be considered. The supraclavicular approach is one option for central venous catheter insertion, but there is little evidence for its use with ECMO catheters, especially DLC catheters, and its safety and feasibility are unknown. Herein, we describe a case of successful DLC placement via a supraclavicular approach.

## Case presentation

A 64-year-old male patient with mantle lymphoma, which was in remission following autogenous blood transplantation, was hospitalized for 39 days for coronavirus disease 2019 (COVID-19) lung injury and refractory pneumothorax. Seven days after admission, hypoxia developed, and V-V ECMO was required to maintain oxygenation and allow video-assisted thoracic surgery (VATS) for pneumothorax to be performed safely. Initially, a 25-Fr single cannula (HLS Cannulae, Maquet Cardiopulmonary GmbH) was inserted via the right IJV for drainage, and a 21-Fr single cannula (HLS Cannulae, Maquet Cardiopulmonary GmbH) was inserted via the right femoral vein (FV) for the return using the ultrasound-guided Seldinger technique. The air leakage was treated successfully with VATS, but the patient was unable to be weaned off V-V ECMO because his serum carbon dioxide (CO_2_) concentration could not be controlled. On day 63, the V-V ECMO configuration was temporarily switched to a 25-Fr single cannula via the right FV for drainage and a 25-Fr single cannula (Bio-Medicus NextGen Cannulae, Medtronic, USA) via the left FV for the return to allow future DLC placement via the right IJV. However, a venous thrombosis, which may have been caused by prolonged retention of the initial 25-Fr drainage cannula, was present in the right IJV and prevented DLC placement. The thrombus remained for two months despite aggressive anticoagulant therapy. This situation forced the patient to remain bedridden without active rehabilitation.

**Figure 1:**
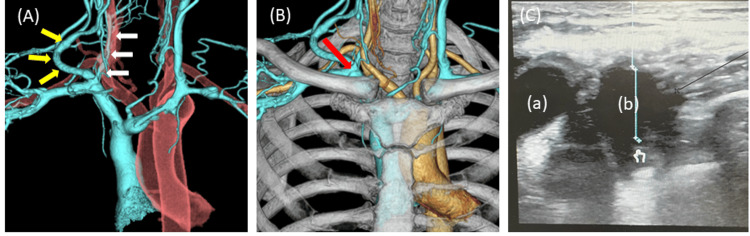
Computed tomography (CT) image taken before the dual-lumen cannula (DLC) insertion (A) A merged CT venography (CTV) and CT angiography (CTA) image of the jugular and thoracic veins and arteries. The yellow arrows indicate a large, well-developed, right external jugular vein functioning as a collateral vessel. The white arrows indicate the right internal jugular vein stenosed by a thrombus. (B) Integration of CTV, CTA, and bones. The red arrow indicates the site where the DLC was inserted. (C) Ultrasound image of the right supraclavicular approach. The right external jugular vein root (a) and the right subclavian vein root (b) are shown. The distance from the skin to the anterior wall of the punctured vessel was only 1 cm.

Computed tomography (CT) on day 104 demonstrated stenosis of the right IVJ due to the thrombus and an enlargement of the right external jugular vein, which functioned as a collateral vessel (Figure 1, Panel A). Therefore, DLC insertion through the IVJ had to be abandoned, although the venous thrombosis in the right IVJ was dissolved. However, the right external jugular vein root was ultrasonographically confirmed through the right supraclavicular approach (Figure 1, Panel C), which ultimately allowed the insertion of the DLC via the supraclavicular approach.

On day 119, after a careful review of CT venography (CTV) findings and ultrasound image, the decision was made to insert a DLC via the supraclavicular approach toward the root of the right subclavian vein (SV) (Figure 1, Panel B). Under fluoroscopic guidance in the operating room and under general anesthesia, a percutaneous approach was made from the supraclavicular area to the root of the right SV with ultrasound guidance. When puncturing the right SV, great care was taken to avoid posterior wall puncture. A hydrophilic guide wire (Radifocus Guide Wire, TERMO, Japan) was introduced into the RA and IVC. Then, serial dilations using 8-Fr, 12-Fr, 16-Fr, 20-Fr, and 24-Fr dilators (Vascular Dilation kit, LivaNava, USA) over the guidewire and blunt dilation of the skin using mosquito pean were carefully performed to firmly dilate the subcutaneous tissue and ensure safe passage of the catheter. After the serial dilations, a 28-Fr DLC (Avalon Elite Bi-caval Dual Lumen catheter, Maquet Cardiopulmonary GmbH) was inserted to the desired depth under fluoroscopic guidance. Transthoracic echocardiography was used to confirm the infusion jet toward the tricuspid valve. Once the DLC was successfully placed and properly connected to the new ECMO circuit, the two cannulae that had been inserted from the FV were clamped and immediately removed.

After the insertion of DLC, no significant changes were observed in the oxygenation and CO_2_ levels. Furthermore, there was no hemodynamic instability during or after DLC insertion. The parameters of vital signs and the performance of the extracorporeal gas exchange circuits before and after the insertion of DLC are shown in Table 1.

**Table 1:** Parameters before and after DLC insertion DLC: Dual-lumen cannula; ECMO: Extracorporeal membrane oxygenation.

		Before DLC insertion on day 119	After DLC insertion on day 120	Day 126
ECMO pump speed	(RPM)	2515	3084	3485
ECMO blood flow	(L/min)	4.0	3.0	3.1
Sweep gas flow	(L/min)	1.5	1.5	1.5
Heart rate	(/min)	94	91	90
Blood pressure	(mmHg)	145/92	152/93	121/80
Respiratory rate	(/min)	16	16	17
PH		7.45	7.47	7.40
PaO_2_	(mmHg)	145	150	124
PaCO_2_	(mmHg)	50	48	58.5
P/F		362	375	310

**Figure 2:**
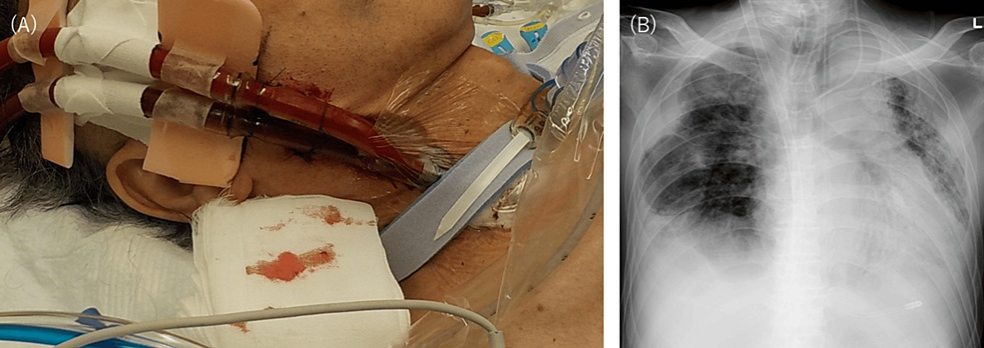
Clinical and X-ray images after the double-lumen cannula (DLC) insertion (A) Cervical image after DLC insertion taken on day 119. The DLC was firmly secured in the right neck without instability or displacement. (B) Chest X-ray after DLC insertion via supraclavicular approach on day 119.

The patient then underwent ECMO treatment with DLC for more than 30 days with no complications such as bleeding or infection. The DLC was firmly anchored in the patient’s right neck without instability or displacement (Figure 2, Panel A), and a chest X-ray confirmed that DLC was not flexed and was naturally implanted (Figure 2, Panel B). Although unable to walk, the patient was able to perform more active rehabilitation in bed, including straight-leg rising and end-sitting, as the catheters were no longer inserted through both thighs. However, the patient ultimately died of unexplained septic shock on day 150.

## Discussion

Critically ill patients usually have many indwelling venous catheters. The insertion sites of these catheters often become unavailable for later use owing to the development of a local infection, hematoma, or thrombosis. The present case, however, involved an atypical case of successful DLC placement for V-V ECMO via a supraclavicular approach, which was chosen because the right IJV was unable to be punctured for anatomical reasons and the presence of a thrombus.

The DLC, which was first used in 2010, has the advantages of requiring only a single venipuncture, allowing greater mobility [1] and reducing the risk of recirculation [2]. However, complications associated with DLC use, including right ventricular rupture and tamponade [3], increased intracranial hemorrhage in cases of large catheter use [4], SVC injury during serial dilation [5], and malpositioning [6], have also been reported.

It is important to note that the DLC is designed to be inserted through the right IJV. While this is done in most cases, there are several other alternative insertion sites, including the left IJV and SV if the right IJV is inaccessible [7]. Shafii et al. proposed the left SV as an insertion site for the DLC, especially for small-stature patients. This is because standard placement of DLC through the IVJ for these patients is particularly troublesome due to excess cannula length. Moreover, the insertion through the left SV may be more comfortable for patient mobility and easier for care from a nursing perspective. It may also reduce the risk of cannula infection when a tracheostomy is present and in close proximity to the jugular vein approach [8]. Memon et al. reported a case series of DLC cannulation via the right SV; the procedure was technically feasible and had a favorable safety profile with a low rate of vascular complications and infection. They also described DLC insertion via the right SV as a practical method for patients requiring long-term V-V ECMO support because it allows greater patient mobility than either jugular or femoral access [9]. However, complications including pneumothorax, hemothorax, and brachial plexus injury are more commonly reported with SV cannulation compared to IVJ [10]. In addition, the IVJ is more superficial and easily accessible, whereas the SV is deeper, which may increase the risk of complications. Thus, although the effectiveness of ECMO cannulation from the SV has been demonstrated, the approach from the right IJV is generally preferred when possible due to the high risk of technical complications and anatomical unfavorability.

Bojic et al. stated that the supraclavicular approach to the SV may be a viable option if the right IJV or FV is not easily accessible. In their prospective analysis of the supraclavicular approach in ECMO cannulation [11], patients underwent supraclavicular cannulation; five involved the femoral-supraclavicular configuration, and six involved DLC insertion via the supraclavicular approach (right: n = 3; left: n = 3). Overall, no insertion-related complications occurred, and no bleeding was observed after cannula removal. The percentage of surviving weaned off from ECMO was similar between the jugular group and the supraclavicular group (79% vs. 73%, respectively) [11]. However, the DLC sizes used were 22 and 24 French (Novalung, Heilbronn, Germany). To the best of our knowledge, no reports have described the use of a 28-Fr DLC yet.

In the present case, two options for DLC placement were considered: via the subclavian area into the right SV or via the supraclavicular area into the root of the right SV. Possible complications in the supraclavicular approach included dislodgment, instability of cannula fixation, risk of pneumothorax, and compression failure during bleeding. Dislodgement was thought to be less likely via the supraclavicular approach than the subclavian approach because the angle to the SVC in the former is more horizontal. As we expected, the DLC was firmly anchored in the right neck, similar to an ECMO catheter inserted through the regular IJV, which was considered more suitable for the DLC than the left or right SV approach. Furthermore, the distance from the skin to the anterior wall of the punctured vessel as measured ultrasonographically was only 1 cm (Figure 1, Panel C), which presumably allowed for compression hemostasis at the time of removal. However, this was unnecessary as the patient ultimately died. Overall, V-V ECMO was able to be managed for more than 30 days with DLC placement via the supraclavicular approach, and no catheter-related complications were observed.

## Conclusions

DLC has come to be used in V-V ECMO for respiratory therapy in adult cases of respiratory failure to facilitate mobilization. If the DLC cannot be inserted through the right IJV, the insertion should not be abandoned. Instead, careful consideration should be given to assess whether insertion from another site is truly impossible. The supraclavicular approach for DLC insertion may be a viable option when IVJ is unavailable due to thrombosis or anatomical reasons. This case suggests that DLC placement via the supraclavicular approach in V-V ECMO is feasible and safe, but further case reports and prospective studies are expected to elucidate the validity of this topic.
